# Nutritional therapy in patients with sepsis: is less really more?

**DOI:** 10.1186/s13054-020-02949-9

**Published:** 2020-05-25

**Authors:** Marie-Sophie Louise Yvonne de Koning, Florianne Johanna Louise van Zanten, Arthur Raymond Hubert van Zanten

**Affiliations:** 1 Department of Cardiology, University Medical Center Groningen, University of Groningen, Groningen, The Netherlands; 2 Amsterdam University Medical Center, University of Amsterdam, Amsterdam, The Netherlands; 3 Department of Intensive Care Medicine, Gelderse Vallei Hospital, Willy Brandtlaan 10, 6716 RP Ede, The Netherlands

Dear Editor,

In a recent review, Van Niekerk and coworkers state permissive underfeeding in septic patients might be beneficial and suggest to investigate the benefits of delayed nutrition in sepsis [1]. They hypothesize that inflammation in sepsis antagonizes the gastrointestinal function in order to sustain catabolism. Persistent catabolism, in turn, might evoke (patho)physiological mechanisms, like autophagy, to ultimately promote cell survival, resulting in outcome benefits. They, therefore, suggest considering the concept of “early fasting” in sepsis. However, most, if not all, studies were based on studies from non-sepsis patients.

There are some indications that this concept may not be valid. First, fasting may lead to combined deficits in calories, vitamins, trace elements, and, most importantly, proteins.

Our group and others have suggested that protein and caloric intake may result in different effects on outcome, particularly concerning the timing of proteins and calories. Retrospective data in general ICU patients suggests early (< day 4) high protein intake may be harmful, possibly by inducing an autophagy-deficient phenotype, while caloric intake might be less important [2, 3]. However, we should be cautious about extrapolating these observations to patients with sepsis.

Two studies specifically addressed the effects of proteins and calories in patients with sepsis during the early phase. In the PROCASEPT study (*n* = 297), we did not observe associations between early (days 1–3) protein and caloric intake and 6-month mortality [4]. This suggests low protein intake or caloric restriction may not be of benefit in sepsis patients. Strikingly, overfeeding during days 4–7 was associated with lower 6-month mortality, compared with low caloric intake. Moreover, Weijs and coworkers were unable to demonstrate an association between caloric and protein intake and outcome in the early phase (day 4) among 127 septic ICU patients [5]. These findings are in contrast with the hypothesis by Van Niekerk and coworkers.

Although retrospective studies are hypothesis-generating, the results of both studies do not suggest early fasting in sepsis might be beneficial. As higher caloric intake and higher protein dosages in the second part of the first week are suggested to benefit the outcome of critically ill patients, a starvation strategy may delay the time to achieve the target and reduce the chance to have the full potential of nutritional therapy during this later phase. We strongly recommend not to implement this starvation hypothesis in sepsis before strong evidence from prospective studies is available.

## Authors’ response

Gustav van Niekerk, Charné Meaker, Anna-Mart Engelbrecht

Dear Editor,

De Koning et al. refer to a number of studies that they claim provide an empirical challenge to the argument we outlined [4]: that permissive underfeeding (PU) during sepsis could hold therapeutic benefit during sepsis. Addressing their concerns, we firstly point out that the key studies they refer to are based on sepsis patients receiving mechanical ventilation (MV). This may suggest that these patients represent uniquely severe cases of sepsis or at least a disease that is quite progressed or complicated. Indeed, one of the studies that they referred to [5] reported a strikingly high mortality rate in the sepsis cohort (48.7%), supporting the notion that the cohort was experiencing a progressed disease. Thus, these patients may not accurately represent the general cohort of sepsis. We also wonder if such a high mortality rate may partially mask any statistical signal (because a large fraction of these patients would die regardless of the intervention, decreasing the “effective” sample size).

Furthermore, infections may have begun days/weeks prior to the study intervention, with the patients’ condition deteriorating progressively. We speculate that a fraction of these patients may have received intermittent/inconsistent nutritional support through the progression from early infection, days/weeks prior to the point where MV was initiated. Also, in the same study [5], it was concluded that “[i]n septic patients, early high protein intake had no beneficial effect on mortality”—i.e., the claim regarding early PU is neutral.

Another study [3] cited by de Koning et al. reported that a low protein intake (< 0.8 g/kg) for the first 3 days, followed by an increase to > 0.8 g/kg protein, was superior to either a chronic high or low protein intake. This does not negate the possibility that PU is beneficial, but perhaps rather indicate that timing the engagement of nutritional support may be more advantageous. We also wonder: would zero initial protein intake perhaps provide even greater benefit than < 0.8 g/kg?

Finally, in another study cited [6], the conclusion was that, in septic patients, “late medium protein and late high energy intake were associated with survival benefit.” Again, this does not indicate that PU is detrimental but suggests that nutritional support at a later stage (during the resolution phase?) may be essential.

We believe these studies highlight the need to investigate markers of disease progression that may be used to map the most appropriate time for the initiation of nutritional support in sepsis.

## Data Availability

Not applicable.
